# Serum Lipids, Apolipoproteins, and Mortality among Coronary Artery Disease Patients

**DOI:** 10.1155/2014/709756

**Published:** 2014-05-29

**Authors:** Ding Ding, Xinrui Li, Jian Qiu, Rui Li, Yuan Zhang, Dongfang Su, Zhongxia Li, Min Wang, Xiaofei Lv, Dongliang Wang, Yan Yang, Min Xia, Dan Li, Gang Hu, Wenhua Ling

**Affiliations:** ^1^Guangdong Provincial Key Laboratory of Food, Nutrition and Health, Department of Nutrition, School of Public Health, Sun Yat-sen University, No. 74 Zhongshan Road 2, Guangzhou, Guangdong 510080, China; ^2^Chronic Disease Epidemiology Laboratory, Pennington Biomedical Research Center, 6400 Perkins Road, Baton Rouge, LA 70808, USA; ^3^Department of Cardiology, General Hospital of Guangzhou Military Command of People's Liberation Army, Guangdong 510010, China

## Abstract

The proatherogenic effect of low-density lipoprotein cholesterol (LDL-C) and antiatherogenic effect of high-density lipoprotein cholesterol (HDL-C) have been confirmed in general population. But controversy arises among coronary artery disease (CAD) patients. The goal of this study was to identify the association of different lipid measurements with CAD prognosis. The study cohort included 1916 CAD patients who were 40–85 years of age. Cox proportional hazards regression models were used to estimate the association of baseline 6 lipid factors and 3 ratios with all-cause and cardiovascular (CVD) mortality. During a median follow-up of 3.1 years, 147 deaths were recorded, 113 of which were due to CVD. When lipid factors were categorized, HDL-C showed a U-shape association with all-cause and CVD mortality after adjustment for major CVD risk factors. Serum LDL-C, apoB, LDL/HDL ratio, and apoB/apoA-I ratio were positively, and apoA-I level was inversely associated with the risk of CVD mortality. After further pairwise comparison of lipid-related risk, LDL/HDL ratio and LDL-C had stronger association with all-cause and CVD mortality than other proatherogenic measurements among Chinese CAD patients.

## 1. Introduction

Cardiovascular disease (CVD) is the leading cause of death worldwide, and it is expected to sharply increase the disease burden over the next 10 years [1]. Lipid metabolism disorder is proven to be associated with the pathogenesis of atherosclerosis, which is fundamental to the occurrence of CVD. Globally, a third of coronary artery disease (CAD) is attributable to high cholesterol, especially low-density lipoprotein cholesterol (LDL-C) [2]. Based on strong epidemiological evidence on the relationship between high LDL-C and an increased risk of CVD, treatment and control of elevated LDL-C are as primary goals of CVD prevention in guidelines [3–5]. In the meantime, both epidemiological and experimental studies confirm the protective effect of high-density lipoprotein cholesterol (HDL-C) on the onset of CAD despite LDL-C level, owing to the reverse cholesterol transport process of HDL-C [6–9]. However, in recent decades, some researchers assert that other newer lipid measurements, including non-HDL-C, apolipoprotein (apo)A-I, apoB, and lipid ratios, are superior to traditional LDL-C in predicting adverse outcomes in general population. Some researchers even suggest that apoB can replace the standard “lipid profile” as a target for motoring and therapy in at-risk patients [10–12]. Besides, several translational studies find that the endothelial effect of HDL-C may be totally different in patients with various clinical conditions [13–15]. Thus, the association between various lipid measurements and secondary risk of CAD deserves more attention due to limited and inconsistent results of previous studies.

Traditionally, the mortality of CAD in China was only one-tenth of that in North America and Australia. A low mean serum total cholesterol (TC) level, associated with a low dietary intake of fat and cholesterol, was considered as the primary reason for the low CAD mortality in China [16]. However, with economic growth and associated lifestyle change in China, Chinese population is experiencing rapid increase in serum lipid levels, while the levels have decreased in most high-income western populations during the past several decades. Raised TC, with up to 33% prevalence among adults aged 25 and above in China, is a primary cause of disease burden among the Chinese population as a risk factor for CVD [2, 17, 18]. Moreover CVD has become the leading cause of death in China [19]. Thus, the aim of the present study was to assess the associations of different lipid measurements with all-cause and CVD mortality among a cohort of Chinese CAD patients and compare their prognostic significance.

## 2. Materials and Methods

### 2.1. Participants

The recruitment of the Guangdong Coronary Artery Disease Cohort was between October 2008 and December 2011 [20]. We enrolled 1980 successive eligible patients admitted to the Cardiology Department of 3 superior specialty hospitals in Guangdong and diagnosed as CAD (International Classification of Diseases- (ICD-) 10 codes I20-I25) according to World Health Organization 1999/2000 guidelines [21, 22]. After excluding 64 participants because of missing data, the final sample comprised 1916 CAD patients aged 40 to 85 years. No differences in age (63.7 versus 62.0 years old, *P* = 0.25), male percentage (65.2% versus 76.6%, *P* = 0.06), and body mass index (BMI) (23.9 versus 23.6, *P* = 0.56) were found between retained participants and excluded participants. The study was approved by Sun Yat-sen University ethnic committee and all clinical investigations were conducted according to the principles expressed in the Declaration of Helsinki, and all participants signed the informed consent.

### 2.2. Clinical Measurements

A standardized questionnaire on general information, including age, gender, education, and marriage status, and a validated food frequency questionnaire [20, 23] were conducted through a face-to-face interview. Smoking was defined as at least one cigarette a day and lasting more than six months. Alcohol drinking was defined as drinking any type of alcoholic beverage at least once a week and lasting more than six months. Smoking and drinking status were classified as never, past, or current [24]. According to the questionnaire, the patients were asked about the frequency and the duration of physical activities in leisure time. Then we calculated the duration of exercise per day.

Clinical characteristics, history of disease, clinical tests' results, and treatment of participants were collected from an electronic case record system. At admission, trained nurses measured height, weight, and blood pressure using a standard protocol [25]. BMI was calculated by dividing weight in kilograms by the square of height in meters. Glomerular filtration rate (GFR) was used to assess renal function which was estimated with the most recent Modification of Diet in Renal Disease Study equation for standardized serum creatinine [26], which is GFR = 175 × (standardized  serum  creatinine  in  mg/dL)^−1.154^ × Age^−0.203^ × 0.742 (if female). Severity of CAD was based on coronary artery stenosis degree of coronary angiography, which was categorized as not conduct, <50%, 50–74.9%, and ≥75%. Treatment information included percutaneous coronary intervention and coronary artery bypass graft. Venous blood samples were drawn in the next morning after hospital admission with at least 12 hours fasting. Lipids and fasting plasma glucose were determined by standard methods. All lipids including TC, triglyceride, LDL-C, HDL-C, apoA-I, and apoB were measured by colorimetric assays using the Hitachi automatic analyzer 7600-020 (Hitachi, Tokyo, Japan). Non-HDL-C levels were calculated by subtracting HDL-C levels from TC levels. Ratios were calculated for LDL/HDL, TC/HDL, and apoB/apoA-I.

### 2.3. Prospective Follow-Up

Follow-up data were collected from hospitals' medical records of readmission, telephone contacts with patients or family members, and death registration of Guangdong Provincial Centers for Disease Control and Prevention. The surveys were followed to the end of July 2013 or patients' death, whichever occurred first. The ICD codes were used to code the cause of death, and the ICD codes I00-I99 were classified as CVD deaths.

### 2.4. Statistical Analysis

Differences in risk factors at baseline by gender were analyzed by the general linear model. The associations between baseline lipid variables and the risks of all-cause and CVD mortality were analyzed by Cox proportional hazards models. The lipid variables were evaluated in the following 2 ways: (1) as categories (<40, 40–49, 50–59, 60–69, ≥70 mg/dL for HDL-C; <70, 70–99, 100–129, 130–159, 160–189, ≥190 mg/dL for LDL-C; <90, 90–109, 110–129, ≥130 mg/dL for apoB; and quartiles for apoA-I, LDL/HDL ratio, and apoB/apoA-I ratio) and (2) as continuous variables (using per 10 mg/dL as a unit). The proportional hazards assumption in the Cox model was assessed with graphical methods and with models including time-by-covariate interactions. In general, all proportionality assumptions were appropriate. To investigate the rank of the lipid variables in terms of strength of association with mortality, their corresponding hazard ratios (HRs) were first determined individually. To directly compare the association of these variables with mortality, pairs of measurements were subsequently included in the model. All analyses were adjusted for age and gender, education, marriage, leisure-time physical activity, smoking, alcohol drinking, and further for types (acute and chronic), severity, duration, treatment of CAD, history of diabetes and heart failure, BMI, systolic blood pressure, GFR, and use of antihypertensive, antidiabetic, and antiplatelet drugs and then additionally for use of cholesterol-lowering drugs. Statistical significance was considered to be 2-sided *P* < 0.05. All statistical analyses were performed with PASW for Windows, version 20.0 (IBM SPSS Inc., Chicago, IL).

## 3. Results

Baseline characteristics of survivors and nonsurvivors were presented in Table 1. Mean age of the population was 63.7 years. Compared with survivors, nonsurvivors were younger, included more females, and had lower levels of fasting plasma glucose and LDL/HDL ratio and high HDL and apoA-I. During a median follow-up of 3.1 years, 147 deaths were recorded, 113 of which were due to CVD. Since the interactions between gender and lipids levels on the risks of all-cause and CVD mortality were not statistically significant, data for men and women were combined in the analyses to maximize the statistical power.

### 3.1. Relationships of Categorical Lipids with Mortality

There was a U-shape association between HDL-C and the risks of all-cause and CVD mortality (Table 2). After adjustment for all confounding factors, CAD patients with HDL-C level below 40 mg/dL had a 2.09-fold risk of all-cause mortality (95% confidence intervals (CI) 1.37–3.20) and a 2.59-fold risk of CVD mortality (95% CI 1.53–4.38), and CAD patients with HDL-C level above 70 mg/dL had a 3.06-fold risk of all-cause mortality (95% CI 1.08–8.70) and a 4.83-fold risk of CVD mortality (95% CI 1.47–15.8), compared with subjects having HDL-C level between 40 and 49 mg/dL. There was a positive association of LDL-C and LDL/HDL ratio with the risk of CVD mortality. Compared with CAD patients having LDL-C level below 70 mg/dL, those with LDL-C level above 190 mg/dL had a 4.35-fold risk of CVD mortality (95% CI 1.34–14.2) after adjusting all confounding factors. CAD patients with the highest quartile of LDL/HDL ratio had the multivariable-adjusted HRs of 1.61 (95% CI 1.01–2.56) for all-cause mortality and 1.74 (95% CI 1.02–2.96) compared with CAD patients with the lowest quartile of LDL/HDL ratio.

The multivariable-adjusted HRs across quartiles of apoA-I were 1.00, 0.59 (95% CI 0.38–0.92), 0.43 (95% CI 0.26–0.70), and 0.56 (95% CI 0.35–0.88) for all-cause mortality (*P* for trend = 0.004) and 1.00, 0.55 (95% CI 0.33–0.91), 0.39 (95% CI 0.22–0.70), and 0.57 (95% CI 0.34–0.95) for CVD mortality (*P* = 0.007), respectively (Table 3). There were significant positive associations of apoB and apoB/apoA-I ratio with the risk of CVD mortality. CAD patients with high level of apoB (≥110 mg/dL) had a 2.06-fold risk of CVD mortality (95% CI 1.10–3.85) compared with CAD patients with low level of apoB (<90 mg/dL). Similarly, CAD patients with the highest quartile of apoB/apoA-I ratio had the multivariable-adjusted HRs of 2.00 (95% CI 1.21–3.31) for all-cause mortality (*P* for trend = 0.058) and 2.33 (95% CI 1.32–4.14) for CVD mortality (*P* for trend = 0.023) compared with CAD patients with the lowest of quartile of apoB/apoA-I ratio.

We further analyzed the concentrations of TC, non-HDL-C, and TC/HDL ratio with the risks of all-cause and CVD mortality and found that CAD patients with high levels of TC (200–239 mg/dL) and non-HDL-C (160–189 mg/dL) were significantly associated with an increased risk of CVD mortality compared with CAD patients with low levels of TC (<150 mg/dL) and non-HDL-C (<100 mg/dL), respectively (Table 4).

### 3.2. Direct Pairwise Comparisons of the Relationships of Continuous Lipids with Mortality

To further compare the strengths of the association of different lipids with all-cause and CVD mortality directly, continuous variables were introduced separately and then in a pairwise mode into the multivariable-adjusted model (Table 5). First, the single measurements were compared with each other. When two antiatherogenic measurements were included simultaneously, apoA-I kept its negative association with all-cause mortality (HR 0.91, 95% CI 0.83–0.99), while HR of HDL-C turned from below 1.00 to above 1.00. When LDL-C was entered into the model with TC or non-HDL-C, the positive relationship between LDL-C and mortalities became significantly stronger, whereas the HRs of TC and non-HDL-C declined a lot to below 1.00. When TC and non-HDL-C were entered into the same model, the HRs for non-HDL-C increased and HRs for TC decreased, although the association remained statistically insignificant. When apoB was included in models with other three cholesterol measurements, all the HRs remained similar. Subsequently, the single proatherogenic measures were directly compared with the ratio variables. Given the inferiority of the relationship of TC and non-HDL-C from the previous comparisons, these two measurements were excluded from the following comparisons. In the analyses, the positive association between LDL/HDL ratio and CVD mortality was stronger when entered into models with LDL-C or apoB, whereas the HRs of LDL-C and apoB both decreased to below 1.00. However, when apoB/apoA-I ratio was included with LDL-C or apoB synchronously, none of the HRs changed. Finally, when we included the two ratio variables in the model, there was little influence on the HRs.

## 4. Discussion

The present study found that high levels of LDL-C, apoB, LDL/HDL ratio, and apoB/apoA-I as well as low level of apoA-I were associated with an increased risk of CVD mortality among Chinese CAD patients, while HDL-C was related to the risk of CVD morality with a U-shape. Moreover, LDL-C and apoA-I were more closely associated with all-cause and CVD mortality than other single measurements, and LDL/HDL ratio showed the greatest statistical association with mortality among other single measurements or ratio.

The low level of HDL-C has been shown to be associated with an increased risk of CAD by many epidemiological and clinical studies [7, 8, 27, 28]. In the Framingham Heart Study, the incidence of coronary events among people with HDL-C below 40 mg/dL was twice high compared with other people [6]. Even among subjects with LDL-C below 70 mg/dL or TC level below 200 mg/dL low HDL-C remains a significant high CVD risk [6, 29]. Moreover, recent evidence has shown that the risk of coronary death decreases 6% with each 1 mg/dL increasing of HDL-C, independent of LDL-C [30]. Thus new strategies for CVD prevention have identified HDL-C as a potential target for therapeutic modification [31, 32]. However, Angeloni et al. found that the protective role of HDL-C was lost in a cohort of CAD patients undergoing elective coronary artery bypass grafting [33]. The recent translational studies demonstrate that the vascular effects of HDL-C may be highly heterogeneous in different clinical conditions. Originally, the antiatherogenic effect of HDL-C isolated from healthy people is related to reverse macrophage cholesterol transport and, more recently, antioxidant and anti-inflammation function which may warrant endothelial homeostasis through stimulating nitric oxide production and inhibiting endothelial apoptosis [34, 35]. But HDL-C isolated from CAD patients or acute coronary syndrome loses its endothelial anti-inflammation capacity and cannot induce endothelial repair due to lack of stimulation of endothelial nitric oxide production. One explanation of this dysfunctional HDL-C is that the critical enzymes which can protect HDL-C from oxidation may be deregulated owing to the oxidizing milieu in CAD patients [14, 36]. Although the exact mechanism has not been clarified yet, this aspect deserves more attention, since therapies of raising HDL-C are in high demand for the secondary prevention of CAD nowadays. In the present study, CAD patients with low (<40 mg/dL) and high (≥70 mg/dL) levels of HDL-C have significantly higher risk of all-cause and CVD mortality than those with HDL-C between 40 and 49 mg/dL, which suggested that the inverse relationship between HDL-C and CVD mortality in general population may be overturned in CAD patients. Thus, our finding strengthens the notion that increasing dysfunctional HDL-C level may be potentially harmful for CAD patients and improving HDL functionality may be more promising [37, 38].

ApoA-I, as the major component and functional protein of HDL, can promote cholesterol efflux from tissues to the liver for excretion from the body. In our study, CAD patients with high apoA-I levels showed a clear trend towards decreased all-cause and CVD mortality. Moreover, apoA-I showed a stronger relationship to mortality than HDL-C in direct comparison, which is consistent with Moss et al.'s study [39]. In their cohort, which enrolled 1045 patients with myocardial infarction, low apoA-I level contributed independently to recurrent coronary events, while HDL-C did not show any significant association with recurrent coronary events.

With regard to various proatherogenic lipid measurements in relation to risk of all-cause and CVD mortality, current researches consist mostly of a series of epidemiological studies in general population. As for secondary prevention study, studies are limited and comprised primarily clinical trial of lipid-lowering drugs with inconsistent conclusions ranging from a strongly positive association to no association at all, because there is no uniformity in data obtained in large epidemiological studies [39–41]. Until now, only one cohort study in Rochester assessed the association of several single lipid measurements with recurrent coronary events and found that only apoB but no other lipid markers (TC and LDL-C) was identified as a predictor for recurrent coronary events during 26-month follow-up [39]. In our study, CAD patients with LDL-C level ≥190 mg/dL or apoB level ≥110 mg/dL had a significantly increased risk of CVD mortality. This difference may be explained by the different methods of LDL-C measurement. The previous study using the Friedewald formula to indirectly estimate LDL-C has a limitation in assessing LDL-C with CVD event, while we used direct colorimetric assay to measure LDL-C. Other existing studies are mainly clinical trials focusing on relationships between lipid levels and clinical outcomes in CAD patients with statin treatment. Data from the long-term intervention with pravastatin in ischemic disease (LIPID) trial and the MRC/BHF heart protection study showed that reduction of either LDL-C or apoB was associated with a reduction of coronary events, but they did not do the analyses on fatal and nonfatal events separately [42, 43]. Another post hoc analysis from two clinical trials found that on-treatment levels of non-HDL-C and apoB were more closely associated with CVD events than LDL-C and inclusion of the antiatherogenic lipid measurements further strengthened the relationships. However, we found that LDL/HDL ratio and LDL-C had better predictive effects on all-cause and CVD mortality than non-HDL-C and apoB among Chinese CAD patients. Since all patients in these two trials received statin therapy and more than 80% of study population were male, the discordance in our results may be due to different characteristics between our population and theirs. Thus, more studies are needed to confirm our finding of better effectiveness of LDL-C and superiority of LDL/HDL ratio in secondary prognosis of CAD. It seems prudent to consider implementing measurements of apoB and apoA-I into routine clinical practice along with LDL-C and HDL-C before their superiority is proven generally.

The finding from the present study supports the significance of lipid regulation in the secondary prevention of CAD. The proven effective strategy for lipid control in Western populations, including national and local cholesterol education programs; the encouraging movement for healthy lifestyle, especially balanced healthy diets; proper use of statins and other lipid-lowering agents; and population-based regular surveillance of serum cholesterol, should be applied to Chinese populations in order to prevent and control dyslipidemia.

There are some limitations in our present study. First, our subjects were enrolled from hospitals which may bring election bias. In general, inpatients are considered to be having a severer disease status than nonhospitalized people. However, we included both acute CAD patients and those with stable manifestation, and some of them were elective admitted patients with mild status. Thus we can reduce the bias. Second, we cannot completely exclude the effects of residual confounding resulting from measurement error in the assessment of confounding factors or some unmeasured factors.

## 5. Conclusions

Among Chinese CAD patients, both too low and too high levels of HDL-C may increase all-cause and CVD mortality. LDL-C remains as an effective and proper predictor for CAD prognosis, while LDL/HDL ratio can strengthen the prediction. Besides, high levels of apoB, apoB/apoA-I ratio, and low apoA-I level can increase the risk of CVD mortality among CAD patients.

## Figures and Tables

**Table 1:** Baseline characteristics by outcomes among coronary artery disease patients.

Characteristic	All patients	Survivors	Nonsurvivors	*P* for difference
*N* (%)	1916	1769 (65.2)	147 (34.8)	
Male (%)	65.2	64.3	75.5	0.006
Age at baseline (yrs)	63.7	63.0 (0.3)	72.4 (0.9)	<0.001
Body mass index (kg/m^2^)	23.9	23.9 (0.1)	23.6 (0.3)	0.22
Systolic blood pressure (mm Hg)	134	134 (0.6)	131 (2.0)	0.10
Diastolic blood pressure (mm Hg)	76	76 (0.3)	76 (1.1)	0.86
Fasting plasma glucose (mmol/L)	6.48	6.41 (0.06)	7.23 (0.23)	0.001
Total cholesterol (mg/dL)	181	181 (1.0)	181 (3.5)	0.97
LDL-C (mg/dL)	114	114 (0.9)	118 (3.2)	0.28
HDL-C (mg/dL)	42	42 (0.3)	40 (0.9)	0.04
Apolipoprotein A-I (mg/dL)	110	111 (0.6)	104 (2.2)	0.003
Apolipoprotein B (mg/dL)	78	78 (0.1)	80 (0.2)	0.39
LDL/HDL ratio	2.87	2.84 (0.03)	3.18 (0.09)	<0.001
Apolipoprotein B/A-I	0.95	0.96 (0.14)	0.85 (0.48)	0.82
Duration of CAD (yrs)				
First diagnosed CAD (*n* = 1026)				
History of CAD (*n* = 890)	2.32 (0.75–7.47)	2.14 (0.68–7.00)	4.76 (1.03–10.0)	0.006
Married (%)	91.7	92.0	87.1	0.11
Years of education (%)				0.22
≤9	61.2	60.7	70.3	
10–12	20.3	20.5	17.6	
≥13	18.5	18.9	12.2	
Smoking (%)				0.35
Never	60.3	60.0	64.1	
Past	9.0	8.9	10.3	
Current	30.7	31.1	25.5	
Alcohol drinking (%)				0.52
Never	77.8	77.5	82.2	
Past	7.2	7.3	5.6	
Current	15.0	15.2	12.1	
Leisure-time physical activity (%)				<0.001
None	34.6	33.5	55.1	
<30 minutes/day	21.6	21.4	24.6	
≥30 minutes/day	43.9	45.1	20.3	
Type of CAD (%)				0.38
Acute coronary syndrome	57.9	58.2	54.4	
Chronic CAD	42.1	41.8	45.6	
Coronary artery stenosis degree of coronary angiography				0.003
Not conduct	34.6	33.8	44.0	
<50%	13.2	13.8	5.7	
50–74.9%	7.5	7.8	3.5	
≥75%	44.7	44.6	46.8	
GFR (mL/min/1.73 m^2^), (%)				<0.001
≥90	28.5	29.6	14.7	
60–89	48.1	48.5	44.1	
30–59	20.8	19.9	31.5	
15–29	1.9	1.5	6.3	
<15	0.7	0.5	3.5	
History of diseases (%)				
Hypertension	57.2	56.7	63.0	0.14
Diabetes	24.9	24.1	34.7	0.004
Dyslipidemia	28.9	29.5	21.1	0.03
Heart failure	42.4	41.3	55.8	0.001
Use of medication before admission (%)				
Antihypertensive drugs	48.9	48.4	54.9	0.14
ACE inhibitors	14.9	14.8	16.7	0.54
Angiotensin II antagonists	20.2	19.9	23.6	0.29
Calcium antagonists	23.8	23.8	23.6	0.97
*β*-blockers	27.8	28.2	22.9	0.17
Diuretics	8.6	7.8	17.4	<0.001
Antidiabetic drugs	16.5	16.0	22.9	0.03
Lipid-lowering drugs	12.0	12.5	6.9	0.05
Antiplatelet drugs	19.4	18.7	27.8	0.008
Treatment of CAD (%)				
Coronary artery bypass graft	2.1	2.1	2.7	0.61
Percutaneous coronary intervention	50.6	50.5	51.0	0.91

Data are mean (SE) or percentage, except duration of CAD is shown median (lower-upper quartiles); all continuous variables are adjusted for age and gender.

**Table 2:** HRs for all-cause and cardiovascular mortality according to different levels of HDL-C, LDL-C, or LDL/HDL.

Variable	Number of deaths		Person-years	Hazard ratios (95% confidence intervals)					
				All-cause mortality			CVD mortality		
	Total	CVD			Model 1^a^	Model 2^b^	Model 3^c^	Model 1^a^	Model 2^b^
HDL-C (mg/dL)									
<40	89	71	2803	2.18 (1.44–3.31)	2.06 (1.35–3.15)	2.09 (1.37–3.20)	2.80 (1.67–4.68)	2.54 (1.51–4.30)	2.59 (1.53–4.38)
40–49	31	19	1998	1.00	1.00	1.00	1.00	1.00	1.00
50–59	16	13	827	1.46 (0.79–2.68)	1.54 (0.83–2.84)	1.53 (0.82–2.83)	1.98 (0.97–4.04)	2.13 (1.03–4.38)	2.11 (1.03–4.35)
60–69	6	6	265	1.93 (0.79–4.72)	1.93 (0.78–4.79)	1.98 (0.80–4.89)	3.20 (1.25–8.23)	3.30 (1.26–8.62)	3.35 (1.28–8.74)
≥70	5	4	104	2.44 (0.94–6.33)	2.93 (1.04–8.24)	3.06 (1.08–8.70)	3.19 (1.07–9.47)	4.67 (1.44–15.1)	4.83 (1.47–15.8)
*P* for difference				0.002	0.006	0.01	0.001	0.002	0.004
LDL-C (mg/dL)									
<70	15	8	641	1.00	1.00	1.00	1.00	1.00	1.00
70–99	47	36	1556	1.18 (0.66–2.12)	1.22 (0.67–2.23)	1.23 (0.68–2.26)	1.72 (0.80–3.70)	1.82 (0.83–4.00)	1.85 (0.84–4.05)
100–129	37	27	1910	0.85 (0.46–1.55)	0.86 (0.46–1.62)	0.86 (0.46–1.61)	1.18 (0.54–2.61)	1.24 (0.55–2.80)	1.25 (0.55–2.82)
130–159	29	24	1191	1.21 (0.64–2.26)	1.28 (0.67–2.45)	1.33 (0.69–2.55)	1.91 (0.86–4.28)	2.08 (0.91–4.74)	2.15 (0.94–4.92)
160–189	14	13	509	1.21 (0.58–2.53)	1.23 (0.58–2.63)	1.21 (0.57–2.59)	2.15 (0.88–5.22)	2.30 (0.92–5.73)	2.29 (0.92–5.72)
≥190	5	5	189	2.08 (0.75–5.79)	2.46 (0.86–7.05)	2.36 (0.82–6.79)	4.03 (1.30–12.5)	4.51 (1.39–14.6)	4.35 (1.34–14.2)
*P* for trend				0.39	0.27	0.27	0.07	0.052	0.055
LDL/HDL ratio									
Quartile 1	35	25	1470	1.00	1.00	1.00	1.00	1.00	1.00
Quartile 2	31	22	1521	1.04 (0.64–1.69)	1.12 (0.68–1.85)	1.10 (0.67–1.82)	1.03 (0.58–1.84)	1.09 (0.60–1.97)	1.08 (0.60–1.95)
Quartile 3	34	27	1483	1.10 (0.69–1.78)	1.05 (0.64–1.71)	1.02 (0.62–1.67)	1.23 (0.71–2.13)	1.11 (0.63–1.95)	1.09 (0.62–1.92)
Quartile 4	47	39	1523	1.67 (1.07–2.60)	1.61 (1.01–2.56)	1.61 (1.01–2.56)	1.91 (1.15–3.19)	1.73 (1.02–2.94)	1.74 (1.02–2.96)
*P* for trend				0.08	0.14	0.13	0.037	0.14	0.12

^a^Model 1 was adjusted for age, gender, education, marriage, leisure-time physical activity, smoking, and alcohol drinking.

^b^Model 2 was adjusted for model 1 covariates plus type, severity, duration, and treatment of CAD, history of diabetes, history of heart failure, BMI, systolic blood pressure, glomerular filtration rate, and use of antihypertensive drugs, antidiabetic drugs, and antiplatelet drugs.

^c^Model 3 was adjusted for model 2 covariates plus use of cholesterol-lowering drugs.

**Table 3:** HRs for all-cause and cardiovascular mortality according to different levels of apoA-I, apoB, and apoB/apoA-I.

Variable	Number of deaths		Person-years	Hazard ratios (95% confidence intervals)					
				All-cause mortality			CVD mortality		
	Total	CVD		Model 1^a^	Model 2^b^	Model 3^c^	Model 1^a^	Model 2^b^	Model 3^c^
ApoA-I									
Quartile 1	51	41	1255	1.00	1.00	1.00	1.00	1.00	1.00
Quartile 2	34	25	1486	0.57 (0.37–0.88)	0.60 (0.38–0.94)	0.59 (0.38–0.92)	0.53 (0.32–0.88)	0.55 (0.33–0.93)	0.55 (0.33–0.91)
Quartile 3	26	19	1603	0.43 (0.26–0.69)	0.43 (0.26–0.71)	0.43 (0.26–0.70)	0.39 (0.23–0.69)	0.40 (0.23–0.70)	0.39 (0.22–0.70)
Quartile 4	36	28	1653	0.52 (0.34–0.81)	0.54 (0.34–0.86)	0.56 (0.35–0.88)	0.52 (0.31–0.85)	0.55 (0.33–0.93)	0.57 (0.34–0.95)
*P* for trend				0.002	0.004	0.004	0.003	0.008	0.007
ApoB (mg/dL)									
<90	102	77	4301	1.00	1.00	1.00	1.00	1.00	1.00
90–109	30	23	1104	1.33 (0.88–2.00)	1.35 (0.88–2.06)	1.35 (0.89–2.07)	1.36 (0.85–2.17)	1.38 (0.85–2.24)	1.39 (0.86–2.26)
≥110	15	13	593	1.74 (1.00–3.04)	1.80 (1.02–3.19)	1.72 (0.97–3.04)	2.00 (1.09–3.66)	2.13 (1.14–3.97)	2.06 (1.10–3.85)
*P* for trend				0.09	0.07	0.10	0.06	0.04	0.05
ApoB/apoA-I									
Quartile 1	29	21	1591	1.00	1.00	1.00	1.00	1.00	1.00
Quartile 2	37	28	1547	1.43 (0.88–2.34)	1.39 (0.85–2.29)	1.34 (0.81–2.21)	1.50 (0.85–2.66)	1.43 (0.80–2.55)	1.38 (0.77–2.48)
Quartile 3	40	29	1496	1.43 (0.88–2.32)	1.44 (0.87–2.37)	1.42 (0.86–2.34)	1.42 (0.80–2.51)	1.36 (0.76–2.44)	1.34 (0.74–2.41)
Quartile 4	41	35	1362	2.18 (1.34–3.58)	2.06 (1.24–3.41)	2.00 (1.21–3.31)	2.58 (1.47–4.51)	2.38 (1.34–4.23)	2.33 (1.32–4.14)
*P* for trend				0.019	0.046	0.058	0.007	0.037	0.023

^a^Model 1 was adjusted for age, gender, education, marriage, leisure-time physical activity, smoking, and alcohol drinking.

^b^Model 2 was adjusted for model 1 covariates plus type, severity, duration, and treatment of CAD, history of diabetes, history of heart failure, BMI, systolic blood pressure, glomerular filtration rate, and use of antihypertensive drugs, antidiabetic drugs, and antiplatelet drugs.

^c^Model 3 was adjusted for model 2 covariates plus use of cholesterol-lowering drugs.

**Table 4:** HRs for all-cause and cardiovascular mortality according to different levels of TC, non-HDL-C, or TC/HDL-C.

Variables	Number of deaths		Person-years	Hazard ratios (95% confidence intervals)					
				All-cause mortality			CVD mortality		
	Total	CVD		Model 1^a^	Model 2^b^	Model 3^c^	Model 1^a^	Model 2^b^	Model 3^c^
TC (mg/dL)									
<150	43	29	1317	1.00	1.00	1.00	1.00	1.00	1.00
150–199	60	45	2843	0.69 (0.46–1.03)	0.71 (0.45–1.08)	0.71 (0.47–1.08)	0.76 (0.47–1.23)	0.80 (0.49–1.32)	0.81 (0.49–1.32)
200–239	35	31	1325	1.14 (0.72–1.82)	1.29 (0.80–2.07)	1.29 (0.80–2.07)	1.54 (0.91–2.59)	1.80 (1.05–3.10)	1.79 (1.04–3.09)
≥240	9	8	512	0.78 (0.37–1.61)	0.82 (0.39–1.75)	0.78 (0.37–1.67)	1.05 (0.48–2.34)	1.17 (0.51–2.68)	1.14 (0.50–2.60)
*P* for trend				0.083	0.053	0.051	0.034	0.013	0.014
Non-HDL-C (mg/dL)									
<100	27	18	921	1.00	1.00	1.00	1.00	1.00	1.00
100–129	45	34	1581	1.06 (0.66–1.71)	1.15 (0.71–1.88)	1.12 (0.68–1.82)	1.19 (0.67–2.11)	1.32 (0.74–2.38)	1.29 (0.72–2.32)
130–159	32	25	1792	0.67 (0.40–1.13)	0.71 (0.41–1.21)	0.70 (0.41–1.20)	0.79 (0.43–1.46)	0.84 (0.45–1.58)	0.84 (0.45–1.58)
160–189	32	27	1027	1.38 (0.82–2.32)	1.52 (0.89–2.59)	1.49 (0.87–2.55)	1.76 (0.96–3.22)	2.03 (1.09-3.79)	2.00 (1.08–3.74)
≥190	11	9	675	0.88 (0.43–1.80)	0.97 (0.47–2.02)	0.92 (0.44–1.92)	1.09 (0.48–2.45)	1.23 (0.53–2.85)	1.18 (0.51–2.73)
*P* for trend				0.07	0.06	0.07	0.07	0.04	0.04
TC/HDL ratio									
Quartile 1	36	27	1474	1.00	1.00	1.00	1.00	1.00	1.00
Quartile 2	30	21	1482	0.93 (0.57–1.51)	0.93 (0.56–1.54)	0.89 (0.54–1.48)	0.86 (0.48–1.53)	0.82 (0.46–1.49)	0.80 (0.44–1.44)
Quartile 3	34	26	1471	1.08 (0.67–1.73)	1.09 (0.67–1.78)	1.08 (0.66–1.75)	1.10 (0.64–1.89)	1.06 (0.60–1.84)	1.05 (0.60–1.83)
Quartile 4	47	39	1570	1.62 (1.04–2.52)	1.53 (0.96–2.43)	1.53 (0.96–2.43)	1.75 (1.06–2.88)	1.56 (0.92–2.64)	1.56 (0.92–2.65)
*P* for trend				0.061	0.15	0.12	0.035	0.11	0.094

^a^Model 1 was adjusted for age, gender, education, marriage, leisure-time physical activity, smoking, and alcohol drinking.

^b^Model 2 was adjusted for model 1 covariates plus type, severity, duration, and treatment of CAD, history of diabetes, history of heart failure, BMI, systolic blood pressure, glomerular filtration rate, and use of antihypertensive drugs, antidiabetic drugs, and antiplatelet drugs.

^c^Model 3 was adjusted for model 2 covariates plus use of cholesterol-lowering drugs.

**Table 5:** Pairwise comparisons of relationships with all-cause and cardiovascular mortality for lipids, apolipoproteins, or their ratios^a^.

	Hazard ratios^b^ (95% confidence intervals)		Hazard ratios^c^ (95% confidence intervals)	
	All-cause mortality	CVD mortality	All-cause mortality	CVD mortality
Comparisons of single measures				
HDL-C	0.91 (0.77–1.07)	0.98 (0.82–1.18)	1.05 (0.85–1.30)	1.17 (0.88–1.42)
Apolipoprotein A-I	0.92 (0.86–0.99)	0.95 (0.88–1.03)	0.91 (0.83–0.99)	0.92 (0.83–1.02)
				
LDL-C	1.02 (0.98–1.07)	1.06 (1.01–1.12)	1.11 (1.01–1.23)	1.16 (1.04–1.29)
Total cholesterol	1.00 (0.96–1.05)	1.03 (0.98–1.08)	0.92 (0.84–1.01)	0.92 (0.83–1.01)
				
LDL-C	1.02 (0.98–1.07)	1.06 (1.01–1.12)	1.08 (0.97–1.20)	1.15 (1.02–1.28)
Non-HDL-C	1.01 (0.97–1.06)	1.04 (0.98–1.09)	0.95 (0.86–1.05)	0.92 (0.82–1.03)
				
Total cholesterol	1.00 (0.96–1.05)	1.03 (0.98–1.08)	0.91 (0.77–1.07)	0.97 (0.81–1.17)
Non-HDL-C	1.01 (0.97–1.06)	1.04 (0.98–1.09)	1.12 (0.94–1.33)	1.07 (0.88–1.29)
				
LDL-C	1.02 (0.98–1.07)	1.06 (1.01–1.12)	1.03 (0.97–1.09)	1.08 (0.99–1.17)
Apolipoprotein B	1.01 (0.97–1.06)	1.03 (0.98–1.07)	0.99 (0.92–1.07)	0.97 (0.86–1.10)
				
Apolipoprotein B	1.01 (0.97–1.06)	1.03 (0.98–1.07)	1.02 (0.96–1.07)	1.01 (0.95–1.08)
Total cholesterol	1.00 (0.96–1.05)	1.03 (0.98–1.08)	1.00 (0.95–1.05)	1.02 (0.97–1.09)
				
Apolipoprotein B	1.01 (0.97–1.06)	1.03 (0.98–1.07)	1.01 (0.95–1.07)	1.01 (0.94–1.08)
Non-HDL-C	1.01 (0.97–1.06)	1.04 (0.98–1.09)	1.01 (0.95–1.06)	1.03 (0.97–1.10)
				
Comparisons of ratio variables and single measures				
LDL/HDL ratio	1.22 (1.06–1.40)	1.27 (1.09–1.49)	1.31 (1.08–1.58)	1.37 (1.10–1.67)
LDL-C	1.02 (0.98–1.07)	1.06 (1.01–1.12)	0.97 (0.91–1.03)	0.98 (0.92–1.05)
				
LDL/HDL ratio	1.22 (1.06–1.40)	1.27 (1.09–1.49)	1.28 (1.07–1.54)	1.30 (1.07–1.58)
Apolipoprotein B	1.01 (0.97–1.06)	1.03 (0.98–1.07)	0.96 (0.87–1.05)	0.98 (0.89–1.08)
				
Apolipoprotein B/A-I ratio	1.00 (0.93–1.08)	1.00 (0.92–1.09)	1.00 (0.91–1.09)	0.99 (0.86–1.13)
LDL-C	1.02 (0.98–1.07)	1.06 (1.01–1.12)	1.02 (0.98–1.07)	1.07 (1.01–1.12)
				
Apolipoprotein B/A-I ratio	1.00 (0.93–1.08)	1.00 (0.92–1.09)	1.00 (0.92–1.08)	0.99 (0.89–1.11)
Apolipoprotein B	1.01 (0.97–1.06)	1.03 (0.98–1.07)	1.01 (0.97–1.06)	1.03 (0.98–1.07)
				
Comparison of ratio variables				
LDL/HDL ratio	1.22 (1.06–1.40)	1.27 (1.09–1.49)	1.22 (1.06–1.42)	1.29 (1.06–1.55)
Apolipoprotein B/A-I ratio	1.00 (0.93–1.08)	1.00 (0.92–1.09)	0.98 (0.81–1.18)	0.94 (0.54–1.66)

^a^Model was adjusted for age, gender, education, marriage, leisure-time physical activity, smoking, alcohol drinking, type, severity, duration, treatment of CAD, history of diabetes, history of heart failure, BMI, systolic blood pressure, glomerular filtration rate, and use of antihypertensive drugs, antidiabetic drugs, antiplatelet drugs, and cholesterol-lowering drugs.

^b^Every variable was introduced into the model singly.

^c^Every pair of variables was simultaneously introduced into the model.
